# The Dual Activity Responsible for the Elongation and Branching of β-(1,3)-Glucan in the Fungal Cell Wall

**DOI:** 10.1128/mBio.00619-17

**Published:** 2017-06-20

**Authors:** Vishukumar Aimanianda, Catherine Simenel, Cecile Garnaud, Cecile Clavaud, Rui Tada, Lise Barbin, Isabelle Mouyna, Christoph Heddergott, Laura Popolo, Yoshikazu Ohya, Muriel Delepierre, Jean-Paul Latge

**Affiliations:** a Unité des *Aspergillus*, Institut Pasteur, Paris, France; b Unité de Résonance Magnétique Nucléaire des Biomolécules, Institut Pasteur, Paris, France; c Dipartmento di Bioscienze, Universita’degli Studi di Milano, Milan, Italy; d Department of Integrated Biosciences and Molecular Biology, The University of Tokyo, Tokyo, Japan; Washington University School of Medicine

**Keywords:** *Saccharomyces cerevisiae*, cell wall, beta-glucan, remodeling, *Aspergillus fumigatus*

## Abstract

β-(1,3)-Glucan, the major fungal cell wall component, ramifies through β-(1,6)-glycosidic linkages, which facilitates its binding with other cell wall components contributing to proper cell wall assembly. Using *Saccharomyces cerevisiae* as a model, we developed a protocol to quantify β-(1,6)-branching on β-(1,3)-glucan. Permeabilized *S. cerevisiae* and radiolabeled substrate UDP-(^14^C)glucose allowed us to determine branching kinetics. A screening aimed at identifying deletion mutants with reduced branching among them revealed only two, the *bgl2*Δ and *gas1*Δ mutants, showing 15% and 70% reductions in the branching, respectively, compared to the wild-type strain. Interestingly, a recombinant Gas1p introduced β-(1,6)-branching on the β-(1,3)-oligomers following its β-(1,3)-elongase activity. Sequential elongation and branching activity of Gas1p occurred on linear β-(1,3)-oligomers as well as Bgl2p-catalyzed products [short β-(1,3)-oligomers linked by a linear β-(1,6)-linkage]. The double *S. cerevisiae gas1*Δ *bgl2*Δ mutant showed a drastically sick phenotype. An *Sc*Gas1p ortholog, Gel4p from *Aspergillus fumigatus*, also showed dual β-(1,3)-glucan elongating and branching activity. Both *Sc*Gas1p and *A. fumigatus* Gel4p sequences are endowed with a carbohydrate binding module (CBM), CBM43, which was required for the dual β-(1,3)-glucan elongating and branching activity. Our report unravels the β-(1,3)-glucan branching mechanism, a phenomenon occurring during construction of the cell wall which is essential for fungal life.

## IMPORTANCE

The fungal cell wall is essential for growth, morphogenesis, protection, and survival. In spite of being essential, cell wall biogenesis, especially the core β-(1,3)-glucan ramification, is poorly understood; the ramified β-(1,3)-glucan interconnects other cell wall components. Once linear β-(1,3)-glucan is synthesized by plasma membrane-bound glucan synthase, the subsequent event is its branching event in the cell wall space. Using *Saccharomyces cerevisiae* as a model, we identified GH72 and GH17 family glycosyltransferases, Gas1p and Bgl2p, respectively, involved in the β-(1,3)-glucan branching. The sick phenotype of the double *Scgas1*Δ *bgl2*Δ mutant suggested that β-(1,3)-glucan branching is essential. In addition to *Sc*Gas1p, GH72 family *Sc*Gas2p and *Aspergillus fumigatus* Gel4p, having CBM43 in their sequences, showed dual β-(1,3)-glucan elongating and branching activity. Our report identifies the fungal cell wall β-(1,3)-glucan branching mechanism. The essentiality of β-(1,3)-glucan branching suggests that enzymes involved in the glucan branching could be exploited as antifungal targets.

## INTRODUCTION

The fungal cell wall plays an important role in maintaining cell shape and integrity and protects the fungal cells from the internal turgor pressure as well as from the external environment (1, 2). This cell wall has a central core composed of a branched β-(1,3)-glucan to which other structural polysaccharides are bound (3–6). Studies have suggested that interlinking between these different polysaccharides is essential for the strength and flexibility of the cell wall (6, 7).

β-(1,3)-Glucan is synthesized as a linear polymer by a plasma membrane-bound synthase complex using UDP-glucose as the substrate (8–11). Linear glucans are elongated further by Gas/Gel/Phr/Epd proteins (12), which belong to the glycosyl-hydrolase 72 (GH72) family (http://www.cazy.org/). These elongases are glycosylphosphatidylinositol (GPI)-anchored plasma membrane proteins, with a glucosyltransferase activity. They cleave an internal β-(1,3)-linkage in a β-(1,3)-glucan and transfer the cleaved fragment to the nonreducing end of another β-(1,3)-glucan acceptor (12–14). GH72 family enzymes have an essential role in the fungal morphogenesis since their gene deletion leads to inviability or results in generation of mutants with significantly reduced growth (15–18). Introducing the β-(1,6)-linkage on the β-(1,3)-glucan is another remodeling process shown to be performed, *in vitro*, by the GH17 family Bgl/Bgt proteins (19–22). These enzymes cleave glucose units from the reducing end of the β-(1,3)-oligosaccharide, transferring enzyme-bound oligosaccharide to an acceptor β-(1,3)-oligosaccharide, either at C-6 of the nonreducing end or at C-6 of an internal glucose unit. However, corresponding deletion mutants did not show β-(1,3)-glucan branching defects (21, 23, 24) or significant growth problems, suggesting the existence of additional/alternative branching mechanisms.

In our study, we identified β-(1,3)-glucan branching activity associated with *Saccharomyces cerevisiae* Gas1p, in addition to its β-(1,3)-glucan elongating activity. This dual β-(1,3)-glucan elongation and branching activity was seen within the members of the GH72 family from both yeasts and molds having a carbohydrate binding module (CBM), CBM43, in their sequence, as *S. cerevisiae* Gas1p (*Sc*Gas1p) ortholog Gel4p from *Aspergillus fumigatus* also showed β-(1,3)-glucan elongation and branching activity. Although *Sc*Gas1p was responsible for the major branching activity, optimum branching was seen in the presence of both Gas1p and *Sc*Bgl2p, suggesting their cooperativity during β-(1,3)-glucan branching. The double *GAS1 BGL2* deletion mutant showed a sick phenotype, and *A. fumigatus GEL4* deletion is inviable (16), indicating that β-(1,3)-glucan elongation branching is an essential event during fungal cell wall construction.

## RESULTS

### Solubilization of the *S. cerevisiae* cell wall alkali-insoluble (AI) fraction with recombinant *endo-*β-(1,3)-glucanase releases both linear and nonlinear β-(1,3)-oligosaccharides.

**FIG 1:**
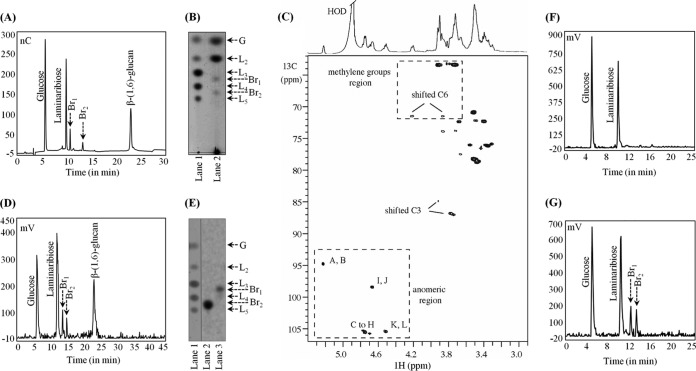
*S. cerevisiae* cell wall β-(1,3)-glucan is β-(1,6)-branched. (A) Dionex profile of the endo-β-(1,3)-glucanase (LamA) solubilized alkali-insoluble (AI) fraction from the wild-type *S. cerevisiae* strain (pulse electrochemical detector [PED], gradient run I). (B) TLC of the LamA-solubilized AI fraction on a silica plate (solvent, ethyl acetate/acetic acid/water [2:1:1]). Lane 1, glucose (G) and laminarioligo standards (L_2_ to L_5_) containing 2 to 5 β-(1,3)-linked glucose units; lane 2, LamA-digested AI fraction, revealed by orcinol-H_2_SO_4_ treatment. (C) ^1^H,^13^C HSQC spectrum of the gel permeation chromatography (GPC)-purified branching oligomers [linear β-(1,3)-oligomers were coisolated due to negligible differences in the *M*_w_; corresponding signals are seen on the HSQC map]. (D) Dionex profile of the LamA-solubilized AI fraction from permeabilized *S. cerevisiae* incubated with UDP-(^14^C)glucose. (E) TLC profile of the GPC-purified ^14^C-labeled branched oligomers (solvent, ethyl acetate/acetic acid/water [2:1:1]). Lane 1, glucose/laminarioligo standards L_2_ to L_5_; lanes 2 and 3, purified radiolabeled branched trimer and tetramer, respectively. All samples were run on the same TLC plate. Lane 1 was separated and revealed by orcinol-H_2_SO_4_ treatment, whereas lanes 2 and 3 were subjected to autoradiography. After the samples were revealed, lane 1 was aligned with lanes 2 and 3 based on the sample application points on the TLC plate before migration. (F) Branching activity is localized in the cell wall. The cytosolic fraction did not incorporate radioactivity upon incubation with UDP-(^14^C)glucose followed by LamA treatment. (G) Membrane fractionation released glucose and laminaribiose, and the cell wall fraction profile showed peaks corresponding to branched oligomers (gradient run I, radiometric detection).

LamA [a recombinant endo-β-(1,3)-glucanase (7, 25)] was employed to solubilize the AI fraction from the wild-type *S. cerevisiae* strain (BY4741). Solubilized material resolved into five major peaks upon high-performance anion-exchange chromatography (HPAEC; Dionex) (Fig. 1A). The first peak and the second peak corresponded to the retention times of glucose and laminaribiose [L_2_, two glucose units joined by a β-(1,3)-glycosidic linkage], respectively, and the peak eluted at the end of the gradient run was β-(1,6)-glucan (7). The elution times of other two peaks (Br_1_ and Br_2_) did not correspond to linear β-(1,3)-oligomers of degrees of polymerization (DPs) of 2 (DP2) to DP6. Upon thin-layer chromatography (TLC), these two peak fractions migrated with *R*_*f*_ values intermediary to those calculated for β-(1,3)-oligotriose-tetraose and β-(1,3)-oligotetraose-pentaose (Fig. 1B). Taken together, these results suggested that two additional peaks did not represent linear β-(1,3)-oligomers. To characterize them, the peak fractions were purified by gel permeation chromatography on a Biogel P2 column and subjected to nuclear magnetic resonance (NMR) analyses.

Figure 1C presents one-dimensional (1D) ^1^H-edited and two-dimensional ^13^C-edited gradient heteronuclear single-quantum correlation spectroscopy (gHSQC) spectra; the anomeric region contained 12 signals corresponding to sugar residues that were arbitrarily labeled A, B, C, D, E, F, G, H, I, J, K, and L. Nearly all the ^1^H and ^13^C resonances could be assigned, although some signals overlapped heavily (see Tables S1 and S2 in the supplemental material). The chemical shifts and ^1^H, ^1^H coupling constant analyses confirmed that all residues corresponded to glucose. The ^3^*J*_H1,H2_ and ^1^*J*_H1,C1_ coupling constant values revealed that all residues except A and B were β-anomers. In the ^13^C-edited gHSQC experiment, two distinct methylene groups can be observed within downfield-shifted H6,6′/C6 at 3.86 to 4.21/71.43 ppm assigned to G and H glucose residues, revealing that both glucose residues were 6-substituted (data in bold in Table S1 in the supplemental material). Furthermore, downfield-shifted C3 carbons characteristic of 3-substituted glucose residues (between 85 and 88 ppm) (Table S1, in bold) were observed for all glucose residues except for the E, F, and L residues identified as non-reducing-end residues from the absence of a chemical shift and for 6-substituted glucose residues (G and H) indicating the absence of disubstituted glucose residue.

10.1128/mBio.00619-17.7 TABLE S1  ^1^H and ^13^C NMR chemical shifts (ppm) and coupling constants ^3^J_H,H_ and ^1^J_C1,H1_ (Hz) for the two additional (branched) oligosaccharides released after digestion of the *S. cerevisiae* AI fraction with endo-β-(1,3)-glucanase (LamA). Download TABLE S1, PDF file, 0.03 MB. Copyright © 2017 Aimanianda et al. 2017 Aimanianda et al. This content is distributed under the terms of the Creative Commons Attribution 4.0 International license.

10.1128/mBio.00619-17.8 TABLE S2  ^1^H and ^13^C NMR chemical shifts (ppm) and coupling constants ^3^J_H,H_ and ^1^J_C1,H1_ (Hz) for the “branched” fraction obtained after β-(1,3)-glucanase digestion of the AI fraction of the wild-type *S. cerevisiae* cell wall. Download TABLE S2, PDF file, 0.02 MB. Copyright © 2017 Aimanianda et al. 2017 Aimanianda et al. This content is distributed under the terms of the Creative Commons Attribution 4.0 International license.

Strong interactions between the anomeric proton (4.510 ppm) of the l-glucose residues and H6/H6′ protons (3.855 to 4.206 ppm) of the G and H glucose residues were observed in the rotating-frame Overhauser effect spectroscopy (ROESY) experiment, corroborated by the presence in the gradient heteronuclear multiple-bond correlation spectroscopy (gHMBC) experiment of an H1/C6 correlation (4.510/71.43 ppm) between these residues, indicating the L_(1 → 6)_G and L_(1 → 6)_H sequence motifs that are β-Glcp-(1→6)-β-Glc*p*-(1→ (data not shown). Dipolar interactions were also observed between the anomeric proton of the G residue (4.704 ppm) and the H3 proton of the I residue (3.734 ppm), suggesting the G_(1→3)_I sequence motif that corresponds to →6)-β-Glcp-(1→3)-β-Glc. This linkage was confirmed by the results of the gHMBC experiment with the observation of the H1/C3 correlation (4.704/88.00 ppm) between these two glucose residues. Similarly, dipolar interactions were observed between the anomeric proton of the H residue (4.687 ppm) and the H3 protons of the A or B residue (3.865 or 3.907 ppm). Moreover, the H1/C3 correlation (4.687/85.89 or 84.81 ppm) between these glucose residues was observed in the gHMBC experiment, indicating the H_(1→3)_A or B sequence pattern that is →6)-β-Glcp-(1→3)-α-Glc. The presence of β-Glc-(1,6)-β-Glc-(1,3)-α,β-Glc, 6-O-branched trisaccharides (Br_1_; Fig. 1A) was thus deduced from these data. In the same manner, NMR data allowed the identification of 6-O-branched tetrasaccharides: β-Glc-(1,3)-β-Glc-(1,6)-β-Glc-(1,3)-α,β-Glc (Br_2_; Fig. 1A).

### Neosynthesis of branched β-(1,3)-glucan by permeabilized *S. cerevisiae*.

Permeabilized *S. cerevisiae* could incorporate radioactivity into the neosynthesized cell wall (7). The LamA-solubilized AI fraction of permeabilized cells incubated with UDP-(^14^C)glucose showed a chromatography profile (Fig. 1D) similar to that from the intact *S. cerevisiae* strain (Fig. 1A). Biogel P2 column-purified neosynthesized nonlinear peaks corresponded to branched β-(1,3)-oligosaccharides by TLC (revealed by autoradiography; Fig. 1E). This result showed that the permeabilized cells could also introduce β-(1,6)-linkages on the linear β-(1,3)-chains. Branched β-(1,3)-oligomers could be detected after 15 min of incubation of the permeabilized cells with UDP-(^14^C)glucose; the branching percentage increased until 1 h, after which it became stationary due to saturation of the enzyme activity. Optimal incorporation was obtained at pH 7.5 and between 25 and 30°C. Removal of ATP, GTP, and EDTA from the buffer reduced glucose incorporation into branching units (data not shown).

Further, *S. cerevisiae* cells were disrupted to separate the cytosolic, membrane, and cell wall fractions and incubated with UDP-(^14^C)glucose individually. There was no radioactivity incorporated with the cytosolic fraction, and the membrane fraction could synthesize only linear β-(1,3)-glucan (Fig. 1F). In contrast, in the cell wall fraction (which contained plasma membrane fragments), branched β-(1,3)-oligomers were detected (Fig. 1G), suggesting that branching occurs in the cell wall after the initial synthesis of linear β-(1,3)-glucan at the plasma membrane.

### Screening of the cell wall-associated gene mutants.

**FIG 2:**
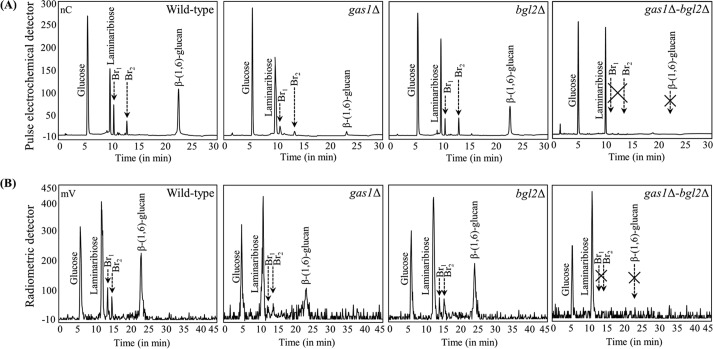
β-(1,3)-Glucan branching was decreased in the *Scgas1*Δ and *Scbgl2*Δ mutants, while the *Scgas1*Δ *bgl2*Δ mutant was devoid of branching on β-(1,3)-glucan. (A and B) Dionex profiles of the LamA-digested cell wall AI fractions from the wild-type strain, single *gas1*Δ mutant, single *bgl2*Δ mutant, and double *gas1*Δ *bgl2*Δ mutant (A) and AI fractions from the corresponding permeabilized cells incubated with UDP-(^14^C)glucose (B) (gradient run II for panel A and gradient run I for panel B).

Deletion mutants involved in the cell wall remodeling were screened for branched β-(1,3)-glucan in their cell walls (Table S3). Upon LamA digestion of the AI fraction followed by Dionex profiling, only two mutants showed a significant difference in the amount of the branched oligomers. As expected, the *bgl2*Δ mutant had a 15% decrease in the amount of branched oligomers. Surprisingly, there was a 70% decrease in the amount of branched oligomers in the *gas1*Δ mutant (Fig. 2A). Branching was unaltered in the mutants involved in the β-(1,6)-glucan biosynthesis. However, a significantly decreased amount of β-(1,6)-glucan in the *gas1*Δ mutant indicated that β-(1,3)-glucan branching is a prerequisite for the β-(1,6)-glucan linkage with the β-(1,3)-glucan. Comparable results were obtained when permeabilized *gas1*Δ and *bgl2*Δ mutant strains were incubated with UDP-(^14^C)glucose and tested for the presence of neosynthesized branched oligomers (Fig. 2B). These data showed that both Gas1p and Bgl2p are involved in the β-(1,6)-branching of β-(1,3)-glucan. To confirm this, a double *gas1*Δ *bgl2*Δ deletion mutant was generated; there was no more branched β-(1,3)-glucan in the cell wall of this double deletion mutant (Fig. 2A), and the corresponding permeabilized cells were unable to neosynthesize β-(1,6)-branched β-(1,3)-glucan (Fig. 2B), suggesting that Gas1p and Bgl2p cooperatively introduce wild-type-level β-(1,6)-branching on the β-(1,3)-glucan.

10.1128/mBio.00619-17.9 TABLE S3  *S. cerevisiae* mutant strains analyzed for branched oligosaccharides. Download TABLE S3, PDF file, 0.02 MB. Copyright © 2017 Aimanianda et al. 2017 Aimanianda et al. This content is distributed under the terms of the Creative Commons Attribution 4.0 International license.

**FIG 3:**
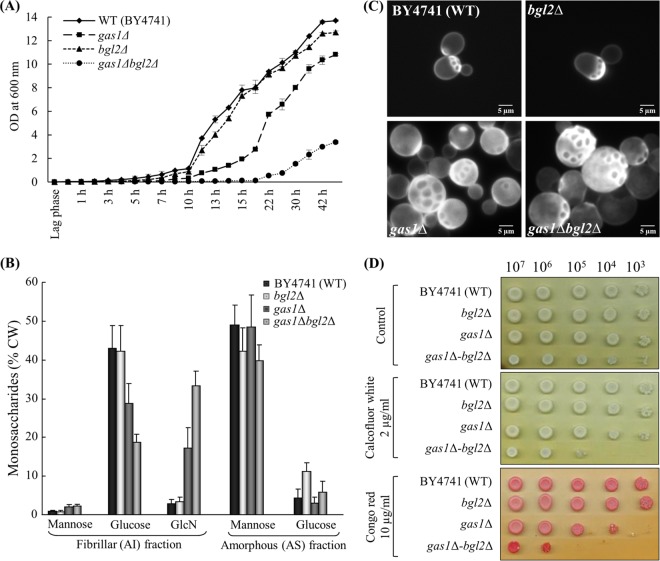
The *gas1*Δ *bgl2*Δ deletion mutant showed a sick phenotype. (A) Growth curves. (B) Cell wall composition. Mannose, glucose, and glucosamine (GlcN) data represent mannan, β-(1,3)-glucan/β-(1,6)-glucan, and chitin content in the cell wall. (C) Calcofluor white (CFW) staining results showing dispersed bud scars and increased labeling intensity with the single *gas1*Δ mutant and the double *gas1*Δ *bgl2*Δ mutant. (D) Sensitivity to cell wall-perturbing agents, CFW, and Congo red (CR). Images were taken after 48 h of growth at 30°C.

In terms of growth, the *bgl2*Δ mutant was similar to the wild-type strain and the *gas1*Δ mutant showed a 20% to 25% decrease, whereas the *gas1*Δ *bgl2*Δ mutant showed a 75% to 80% decrease after 48 h (Fig. 3A). Cell wall analyses of the single *gas1*Δ and the double *gas1*Δ *bgl2*Δ mutant strains showed altered compositions, while the *bgl2*Δ cell wall composition was comparable to that of the wild-type strain (Fig. 3B). The *gas1*Δ and *gas1*Δ *bgl2*Δ mutants showed a significant (2-fold to 8-fold) increase in the amount of cell wall chitin content (represented by GlcN) and a 2-fold decrease in the AI fraction glucose level. The amorphous (AS) fraction compositions were not altered by the single *GAS1* and the double *GAS1 BGL2* gene deletions. Calcofluor white (CFW) staining of the cells was more intense for the *gas1*Δ and *gas1*Δ *bgl2*Δ mutants than for the parental strain, supporting the data indicating increased cell wall chitin levels (Fig. 3C). CFW staining also showed dispersed distribution of bud scars on the *gas1*Δ and *gas1*Δ *bgl2*Δ mutant surfaces, in contrast to the presence of polarized bud scars on the wild-type strain and *bgl2*Δ mutant surfaces, suggesting a disorganized cell wall leading to nonpolarized budding in the *gas1*Δ and *gas1*Δ *bgl2*Δ mutants. In addition, the *gas1*Δ and *gas1*Δ *bgl2*Δ mutant cells were, respectively, 1.5-fold ± 0.3-fold and 1.7-fold ± 0.4-fold bigger than the wild-type strain and *bgl2*Δ mutant cells. Additionally, the *gas1*Δ *bgl2*Δ mutant and, to a lesser extent, the *gas1*Δ mutant were sensitive to cell wall-perturbing compounds such as Congo red and CFW (Fig. 3D).

### Gas1p and Bgl2p are responsible for the branching of the cell wall β-(1,3)-glucan in yeast.

**FIG 4:**
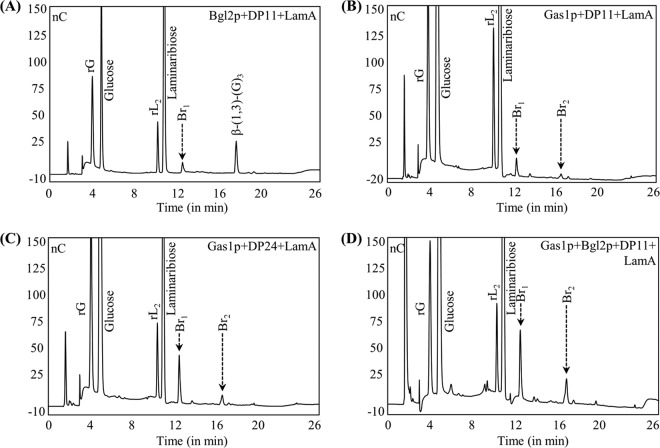
Recombinant Gas1p and Bgl2p showed β-(1,3)-glucan branching activity. β-(1,3)-Oligomer (DP11) was incubated with Gas1p, with Bgl2p, or with both (37°C), followed by LamA digestion and Dionex profiling. (A) Bgl2p+DP11 (24 h) plus LamA addition. (B) Gas1p+DP11 (24 h) plus LamA addition. (C) Gas1p+DP24 (24 h) plus LamA addition. (D) Gas1p+Bgl2p+DP11 (20 h) plus LamA addition (PA1 column, gradient run II; the oligomeric substrates used were reduced [“r”], which released reduced glucose rG and reduced laminaribiose rL_2_ [rG/rL_2_] upon LamA digestion).

As the minimum length of β-(1,3)-oligomers required for Gas1p activity was 11 monomeric units (12), we tested β-(1,3)-oligomers with a degree of polymerization (DP) of 11 and above. Similar to previous reports (21, 26), Bgl2p did introduce a β-(1,6)-linkage on the DP11 oligomer (Fig. 4A). Incubation of Gas1p with the DP11 oligomer initially resulted in the elongation of the β-(1,3)-chain (12), but during the later time course (>20 h of incubation) it introduced β-(1,6)-branching on the reaction products (Fig. 4B). Results of reactions performed with DP15 and DP24 were similar to those seen with DP11, but branching signals were detectable at earlier incubation times with increasing DPs of the oligomeric substrate: the first branching signal was observed when DP24 and DP15 were incubated with Gas1p for 12 h and 16 h, respectively (Fig. 4C, profile for DP24). This result suggested that an increase in the β-(1,3)-oligosaccharide length decreases the time required to introduce β-(1,6)-linkages on them by Gas1p. A medium pH range tested (pH 5.0 to 7.0) had no effect on the Gas1p branching activity. An increase in the branch signal upon coincubation of Gas1p and Bgl2p (Fig. 4D) compared to the results seen with their individual incubation with DP11 confirmed their cooperativity in β-(1,3)-glucan branching activity.

To confirm that the peaks corresponding to branched oligosaccharides obtained in the Dionex profile upon recombinant Gas1p activity on β-(1,3)-oligosaccharides represented bona fide β-(1,6)-branched oligomers, we either analyzed the purified fraction corresponding to the trimer by NMR or, prior to LamA digestion, subjected the Gas1p and β-(1,3)-oligosaccharide reaction mixture to periodate oxidation-Smith degradation [which destroys linear β-(1,6)-linkages]. NMR analysis confirmed the presence of β-(1,6)-linkage in the purified oligosaccharide (see Fig. S1 in the supplemental material; shifted C6 signals indicated the presence of 6-O-branched glucose residues in the analyzed fraction). The sparse quantity of the oligomer isolated and the fact that this oligomeric sample was in natural abundance precluded the assignment of all other cycle ^1^H and ^13^C resonances associated with these shifted C6 signals. Thus, these signals might originate from →6)-β-Glc*p*- and/or →3,6)-β-Glc*p*-. However, the presence of periodate oxidation-Smith degradation-resistant branched oligosaccharides (Fig. S2) confirmed that the β-(1,6)-linkages were →3,6)-β-Glc*p*-.

10.1128/mBio.00619-17.1 FIG S1  NMR analysis confirmed that incubation of Gas1p with β-(1,3)-oligosaccharide resulted in the introduction of a β-(1,6)-linkage. The figure represents a ^1^H, ^13^C HSQC partial spectrum of β-(1,3)-oligomers incubated with Gas1p for more than 30 h. A C6 chemical shift indicates the introduction of a branching on the β-(1,3)-glucan. In order to get better resolution in the methylene region and increase the sensitivity, the components of the HSQC experiment (performed using a Varian BioPack) were folded in the ^13^C dimension. The ^13^C decoupler offset was centered at 70 ppm, and the ^13^C sweep width was reduced to 42 ppm. The experimental data were acquired with 128 increments and 432 accumulations at a temperature of 288 K. Download FIG S1, TIF file, 0.1 MB. Copyright © 2017 Aimanianda et al. 2017 Aimanianda et al. This content is distributed under the terms of the Creative Commons Attribution 4.0 International license.

10.1128/mBio.00619-17.2 FIG S2  Gas1p introduces β-(1,6)-linkages on the β-(1,3)-glucan nonlinearly. The Gas1p+DP11 catalyzed reaction mixture was subjected to periodate oxidation-Smith degradation followed by LamA treatment and Dionex profiling (gradient run II; rG/rL_2_; see Fig. 7). The peaks corresponding to branched oligosaccharide were resistant, confirming that Gas1p nonlinearly introduced β-(1,6)-linkages on the β-(1,3)-oligosaccharides. Download FIG S2, TIF file, 0.1 MB. Copyright © 2017 Aimanianda et al. 2017 Aimanianda et al. This content is distributed under the terms of the Creative Commons Attribution 4.0 International license.

**FIG 5:**
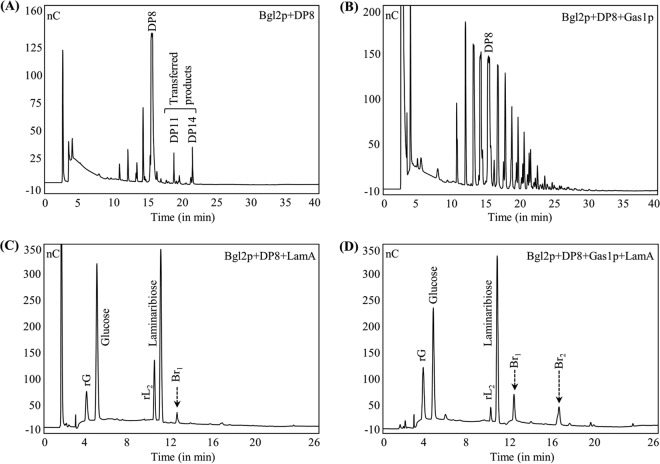
Recombinant Gas1p could utilize transferred Bgl2p products as the substrate. The figure presents Dionex profiles of Bgl2p incubated with β-(1,3)-oligomer of DP8 overnight (A) followed by either addition of LamA and analysis of the sample (B) or by heat inactivation of Bgl2p and addition of Gas1p, a further incubation performed overnight, and direct analysis of the products (C) or analysis performed after LamA treatment (D) (gradient run II and gradient run III for the PA1 [profiles C and D] and PA200 [profiles A and B] columns, respectively; rG/rL_2_ [see Fig. 4]).

The minimum β-(1,3)-oligomer lengths required for Gas1p and Bgl2p activity were 11 and 5, respectively (12, 21). Incubation of β-(1,3)-oligosaccharide of DP8 with Bgl2p resulted in the transferred products of DP that were higher than 8 (Fig. 5A). Upon heat inactivation of Bgl2p followed by the addition of Gas1p to the reaction mixture, products of higher DPs were synthesized (Fig. 5B), suggesting that Gas1p could utilize Bgl2p-catalyzed reaction products. Further, addition of Gas1p to the heat-inactivated Bgl2p-catalyzed reaction mixture resulted in the formation of β-(1,6)-branched oligomers in larger amounts [Fig. 5C and D, representing Dionex profiles of the LamA-solubilized Bgl2p+DP8 and (Bgl2p+DP8) plus Gas1p reactions, respectively], confirming that Gas1p and Bgl2p act cooperatively *in situ* in introducing β-(1,6)-branches on the β-(1,3)-glucan.

### β-(1,3)-Glucan branching activity of recombinant Gel4p from *A. fumigatus*.

**FIG 6:**
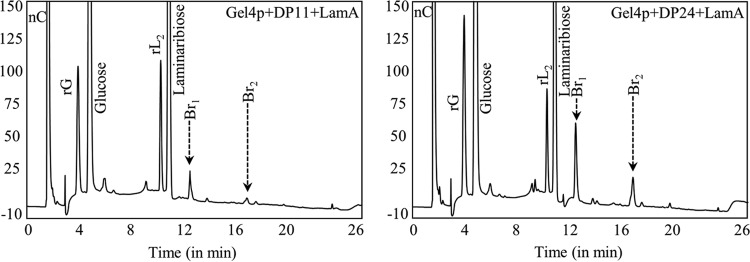
*A. fumigatus* Gel4p also showed β-(1,3)-glucan branching activity. (A) Similar to Gas1p, a recombinant *Af*Gel4p could introduce β-(1,6)-linkages on the β-(1,3)-oligomers of DP11. (B) There was an increase in the amount of β-(1,6)-linkages introduced when DP24 was used as the substrate (gradient run II; rG/rL_2_; see Fig. 4).

The Dionex and TLC profiles of the LamA-digested AI fraction from *A. fumigatus* mycelium and *Candida albicans* (profiles not shown) were similar to those of *S. cerevisiae*; the branching percentages were ~5.8% and ~3.7%, respectively, comparable to that determined for *S. cerevisiae* (4.4%). Having demonstrated that *Sc*Gas1p catalyzes β-(1,3)-glucan branching, orthologous *Af*Gel4p was tested for such activity. Incubated with DP11 for 20 h, *Af*Gel4p was able to introduce β-(1,6)-branching (Fig. 6). Incubation of Gel4p with oligosaccharides of higher DP resulted in an increased branching, and the longer the β-(1,3)-oligosaccharides were, the earlier the β-(1,6)-linkages were introduced (Fig. 6; Fig. S3). Gel4p-introduced β-(1,6)-linkages were also resistant to periodate oxidation-Smith degradation (Fig. S4), suggesting dual elongation and branching activity associated with GH72 family glycosyltransferases from both the yeast *S. cerevisiae* and the pathogenic mold *A. fumigatus*.

10.1128/mBio.00619-17.3 FIG S3  β-(1,3)-oligomeric chain length affects the branching efficiency of *Af*Gel4p. Gel4p was incubated with β-(1,3)-oligomers of DP24 and DP34 followed by LamA treatment and Dionex profiling (gradient run II). Similarly to *Sc*Gas1p, *Af*Gel4p introduced branching at early time points, with increases in the chain length of the substrate (rG/rL_2_) (explained in the Fig. 4 legend; not seen in the Dionex profile with DP34 as it was not reduced, unlike DP24). Download FIG S3, TIF file, 0.1 MB. Copyright © 2017 Aimanianda et al. 2017 Aimanianda et al. This content is distributed under the terms of the Creative Commons Attribution 4.0 International license.

10.1128/mBio.00619-17.4 FIG S4  *Af*Gel4p also introduced β-(1,6)-linkages nonlinearly on the β-(1,3)-oligosaccharide. A Gel4p-DP18-catalyzed reaction mixture was subjected to periodate oxidation-Smith degradation followed by LamA treatment and Dionex profiling (gradient run II). The branched oligosaccharide peaks were resistant, confirming that *Af*Gel4p also introduces β-(1,6)-linkages on the β-(1,3)-oligosaccharides nonlinearly (rG/rL_2_; see Fig. 4). Download FIG S4, TIF file, 0.04 MB. Copyright © 2017 Aimanianda et al. 2017 Aimanianda et al. This content is distributed under the terms of the Creative Commons Attribution 4.0 International license.

### A carbohydrate binding module (CBM), CBM43, is necessary for the GH72 family Gas/Gel proteins to show dual β-(1,3)-glucan elongation-branching activity.

**FIG 7:**
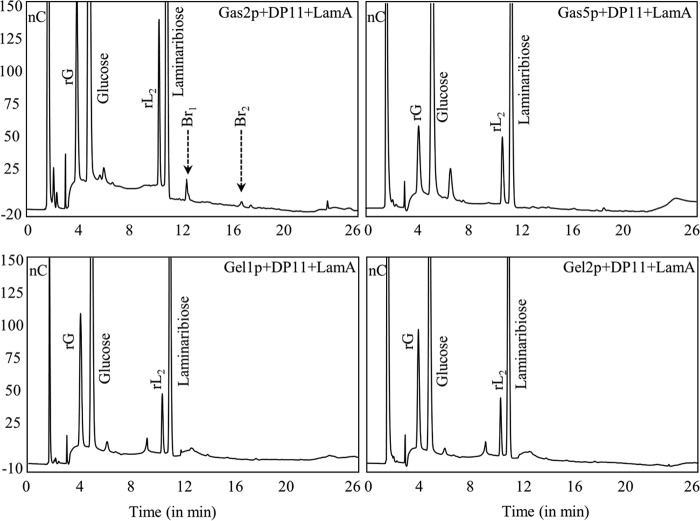
Dual elongating-branching activity was associated with a putative CBM, CBM43. Similar to *Sc*Gas1p and *Af*Gel4p, *Sc*Gas2p, but not *Sc*Gas5p, *Af*Gel1p, or *Af*Gel2p, showed elongating-branching activity. *Sc*Gas1p, *Sc*Gas2p, and *Af*Gel4p, but not *Sc*Gas5p, *Af*Gel1p, and *Af*Gel2p, are characterized by the presence of a CBM (CBM43), suggesting that the dual elongating-branching activity is associated with glycosyltransferases with a putative CBM, CBM43 (gradient run II; rG/rL_2_; see Fig. 4).

*Sc*Gas1p, *Sc*Gas2p, and *Af*Gel4p showed dual β-(1,3)-glucan elongating and branching activity; they belong to the GH72 family and contain a putative CBM (CBM43) at their C terminus. However, *Sc*Gas5p, *Af*Gel1p, and *Af*Gel2p, which also belong to GH72 family but are devoid of CBM43, showed only β-(1,3)-glucan elongase activity (Fig. 7), suggesting that linear elongation of β-(1,3)-glucan is a prerequisite and that the positioning of elongated β-(1,3)-glucan by the CBM43 is absolutely required for the subsequent branching activity of Gas/Gel proteins.

## DISCUSSION

β-Glucan is the major constituent of fungal cell wall, with its amount ranging between 30% and 80% of the cell wall dry mass depending on the fungal species (2). It is a branched polymer with branches attached to the core polymer by β-(1,6)-linkages (27). Branching ramifies β-(1,3)-glucan, facilitating its binding with other cell wall components, and hence it is considered to be essential for the cell wall architecture (3). Until now, β-(1,3)-glucan branching was a mystery, as membrane preparations synthesized only linear β-(1,3)-glucan *in vitro* (7, 9). In the present study, we showed that mature β-(1,3)-glucan biosynthesis requires the presence of both cell wall and membrane fractions and that branching in the yeast *S. cerevisiae* occurs due to cooperative activity of two glycosyltransferases, Gas1p and Bgl2p, previously shown to display a unique genetic interaction (http://www.yeastgenome.org/locus/S000004924/interaction).

The *gas1*Δ mutant, in agreement with earlier reports (28, 29), showed a mild growth defect and altered cell morphology with spherical cells and dispersed bud scars, unlike the wild-type strain, which showed an ellipsoidal shape and bud scars concentrated at one pole. The double mutant also showed enlarged spherical cells with dispersed bud scars. In the *gas1*Δ and *gas1*Δ *bgl2*Δ mutants, there were 90% and 98% decreases in the cell wall β-(1,6)-glucan content, respectively, compared to the wild-type strain. Orlean (6) reported that the *S. cerevisiae* cell wall is organized in the order β-(1,3)-glucan→β-(1,6)-glucan→mannoproteins. However, first, we did not find β-(1,6)-glucan in the *gas1*Δ *bgl2*Δ mutant cell wall, suggesting that the β-(1,3)-glucan ramification is essential for β-(1,6)-glucan attachment. Second, according to the described organization order, mannoproteins must be present in the fibrillar (AI) fraction of the cell wall and the absence of β-(1,6)-glucan must result in the loss of mannoproteins from the cell wall due to the lack of an anchoring structure. However, in our study, we extracted mannan mainly in the amorphous (AS) fraction of the wild-type strain and its amount in the mutants was similar to that in the wild-type strain, indicating that mannan is not covalently bound to the other cell wall components. Magnelli and coworkers were also able to extract mannan in the alkali-soluble (AS) fraction (30), whereas Ballou reported its extraction using citrate buffer (31), which supports our observation and the idea that, as a mannoprotein, mannan occurs as a fibrillar outer layer in the yeast cell wall (32). When the *gas1*Δ *bgl2*Δ mutant culture supernatant was analyzed, we did not find β-(1,6)-glucan, suggesting that β-(1,3)-glucan branching indeed affects β-(1,6)-glucan biosynthesis. There is still a debate about the site and mechanism of β-(1,6)-glucan biosynthesis (33–36). But our study results suggest that the order of cell wall construction is branched β-(1,3)-glucan→β-(1,6)-glucan and reinforce the speculations published by earlier researchers that the maturation of β-(1,6)-glucan occurs in the cell wall (37). Moreover, the lack of β-(1,6)-glucan in the *gas1*Δ *bgl2*Δ mutant cell wall and the fact that the presence of proteins involved the β-(1,6)-glucan biosynthesis in the cell wall and/or plasma membrane-associated forms (38–42) led to the speculation that (i) the biosynthesis and maturation of β-(1,6)-glucan occur in the cell wall and (ii) Kre5p functions as a chaperon for the proteins involved in the β-(1,6)-glucan biosynthesis (36) rather than being involved in the synthesis of nascent β-1,6-glucan chains (42, 43). Double deletion of *GAS1* with genes involved in β-(1,6)-glucan synthesis (*KRE1* or *KRE6*) is synthetically lethal (http://www.yeastgenome.org/), which suggests that β-(1,3)-glucan elongation-branching and β-(1,6)-glucan biosynthesis-attachment to branched β-(1,3)-glucan are essential events during cell wall construction. Double deletion of *GAS1* and *CHS1* or *CHS3* (involved in chitin synthesis) resulted in a lysed-bud or a severely compromised phenotype (44), suggesting that both branched β-(1,3)-glucan and chitin are important in the cell wall. However, no such lethality has been described for the *CRH1 CRH2* double deletion (45) [Crh1p and Crh2p are involved in linking chitin to β-(1,3)-glucan and β-(1,6)-glucan (46, 47), respectively], suggesting that glucan-chitin linkage may not be an essential part of the cell wall fibrillar core.

**FIG 8:**
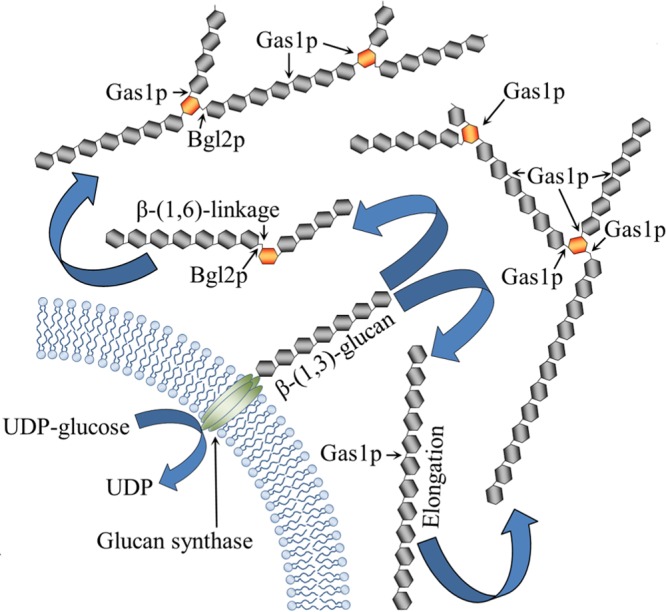
Mechanism of *S. cerevisiae* cell wall β-(1,3)-glucan branching—a model. Short linear β-(1,3)-glucans are synthesized by a plasma membrane-bound glucan synthase complex using UDP-glucose as the substrate. The short linear glucans entering cell wall space undergo further elongation by Gas1p or are linked to another short β-(1,3)-glucan by Bgl2p through a linear β-(1,6)-linkage. Gas1p utilizes self-elongated glucan for branching, or it can elongate a Bgl2p-catalyzed product which contains a free carbon(C)-3 hydroxyl (−OH) group(s) on the β-(1,6)-linked glucose unit. In the following step, Gas1p further elongates and branches β-(1,3)-glucan, resulting in the formation of a ramified β-(1,3)-glucan.

Gas1p and Bgl2p are among the best-characterized glycosyltransferases (48). Our study data have allowed a better understanding of their role. The phenotypes of the single *gas1*Δ and double *gas1*Δ *bgl2*Δ mutants the we analyzed were in agreement with the observations by Plotnikova et al. (48) indicating that Gas1p and Bgl2p are functionally related. Bgl2p is one of the most abundant cell wall proteins and is able to introduce β-(1,6)-linkages on β-(1,3)-glucan (24). However, Bgl2p preferred shorter β-(1,3)-oligomers, as there was a decrease in its activity upon an increase in the length of the oligomeric substrate (see Fig. S5 in the supplemental material). Initially, recombinant Gas1p showed β-(1,3)-elongase activity followed by the introduction of β-(1,6)-linkages on the β-(1,3)-glucan, suggesting that branching activity of Gas1p is dependent on the elongation of the β-(1,3)-glucan chain that generates an appropriate substrate for branching. In support of this hypothesis, with β-(1,3)-oligomers of greater chain length, there was a shorter incubation time before the appearance of branches. There was a significant increase in the branching when Gas1p and Bgl2p were incubated together with β-(1,3)-oligomers, suggesting their cooperative branching activity. Bgl2p preferring shorter β-(1,3)-oligomers and Gas1p elongating β-(1,3)-oligomers prior to its β-(1,6)-branching activity suggest the hypothesized mechanism of branching activity depicted in Fig. 8. Supporting our model, the branching signal seen in the LamA-digested AI fraction from the *gas1*Δ mutant could be destroyed completely upon prior periodate oxidation-Smith degradation of the AI fraction, whereas the AI fractions from the wild-type and *bgl2*Δ mutant strains were resistant to such treatment, with the wild-type strain showing less than 10% destruction of the branched trimer (Fig. S6). This result confirms that, in the wild-type strain, Bgl2p introduces less than 15% of linear β-(1,6)-linkage on the short β-(1,3)-oligomers synthesized by the plasma membrane-bound glucan synthase complex, which could be destroyed by periodate oxidation-Smith degradation. In contrast, in the *gas1*Δ mutant, possibly more short β-(1,3)-oligomers are available for Bgl2p to introduce β-(1,6)-linkages (Fig. 2A; 30% instead of the 10% to 15% branching introduced by Bgl2p in the wild-type cell wall) due to the lack of Gas1p activity that initially elongates shorter β-(1,3)-oligomers synthesized by the glucan synthase complex; as these Bgl2p introduced β-(1,6)-linkages are linear, they could be completely destroyed by periodate oxidation-Smith degradation (Fig. S6).

10.1128/mBio.00619-17.5 FIG S5  Bgl2p prefers short β-(1,3)-oligosaccharides for introduction of β-(1,6)-linkages. Dionex profiles (gradient run II) seen upon incubation of Bgl2p with β-(1,3)-oligomers of DP6, DP11, DP18, and DP24 overnight at 37°C followed by LamA digestion are presented. With an increase in the length of the oligosaccharides from 6 to 18, there was a decrease in the amount of β-(1,6)-linkages introduced; with DP24, there was no additional β-(1,6)-linkage introduced. Download FIG S5, TIF file, 0.1 MB. Copyright © 2017 Aimanianda et al. 2017 Aimanianda et al. This content is distributed under the terms of the Creative Commons Attribution 4.0 International license.

10.1128/mBio.00619-17.6 FIG S6  *S. cerevisiae* cell wall β-(1,3)-glucan is branched. The AI fraction was subjected to periodate oxidation-Smith degradation prior to LamA treatment. The wild-type and *bgl2*Δ strains showed a decrease of less than 10% in the Br_1_ signal on Dionex profiling (branched trimer) compared to the AI fraction subjected directly to LamA treatment (Fig. 1A), whereas that of *gas1*Δ was destroyed completely (gradient run I, pulsed electrochemical detector). Download FIG S6, TIF file, 0.04 MB. Copyright © 2017 Aimanianda et al. 2017 Aimanianda et al. This content is distributed under the terms of the Creative Commons Attribution 4.0 International license.

The dual activity seen in our study with GH72 family fungal glycosyltransferases carrying a CBM is not an exception in biology. Adenylosuccinate lyase from *Thermotoga maritima*, which forms a homotetramer, catalyzes the addition of nitrogen at two different positions of AMP in a reaction involving the beta-elimination of fumarate (49). Its dual activity is attributed to a single 180°-bond rotation in the substrate between the first and the second enzymatic activities. AmiA, a chlamydial enzyme, acts both as a carboxypeptidase and an amidase, the former activity being associated with the presence of a penicillin-binding protein motif (50). A 175-kDa enzyme from *Candida utilis* showed trehalase-sucrase activity (51). In the present study, only those GH72 family members with a CBM showed dual activity, suggesting that proper positioning of the substrate by a CBM is essential. We did attempt to delete the CBM from *GAS* family members; however, such a deletion where a CBM is comprised of 90 to 100 amino acids (http://www.cazy.org/) resulted in the complete loss of both elongating and branching activity, possibly due to the loss of active enzyme structure.

In conclusion, in *S. cerevisiae*, both Gas1p and Bgl2p are involved in the β-(1,6)-branching of the cell wall β-(1,3)-glucan; Bgl2p prefers shorter β-(1,3)-glucan chains, whereas Gas1p acts on self-elongated β-(1,3)-chains as well as on Bgl2p transglycosylated products. The *gas1*Δ *bgl2*Δ deletion mutant was devoid of (i) β-(1,6)-branching on the β-(1,3)-glucan and (ii) β-(1,6)-glucan in the cell wall, indicating that β-(1,6)-glucan biosynthesis occurs in the cell wall and that ramification of β-(1,3)-glucan is necessary for β-(1,6)-glucan biosynthesis. The *Sc*Gas1p ortholog Gel4p from the filamentous fungus *A. fumigatus* also showed dual elongating-branching activity, indicating that the β-(1,3)-glucan branching mechanism is likely to be conserved across the fungal kingdom. The *S. cerevisiae gas1*Δ *bgl2*Δ mutant showed an extremely sick phenotype, and the *A. fumigatus GEL4* deletion was lethal (16), suggesting that β-(1,3)-glucan elongation-branching is an essential process during fungal cell wall construction, and such activity could be exploited as an antifungal target.

## MATERIALS AND METHODS

### Yeast strains and growth conditions.

The *S. cerevisiae* strains used were *BY4741*, mutant *gas1*Δ, and mutant *bgl2*Δ (EUROSCARF; Unité de Génétique Moléculaire des Levures, Institut Pasteur, Paris, France). The rest of the mutants (EUROSCARF collection) were from Yoshikazu Ohya, Tokyo University, Japan. Cells were grown in yeast extract-peptone-dextrose (YPD) medium (2% glucose, 1% Bacto Peptone, and 2% yeast extract) at 30°C and harvested in their early logarithmic-growth phase (optical density at 600 nm [OD_600_], 3 to 4).

### Construction of *gas1*Δ *bgl2*Δ double deletion mutant strain.

Primers used are listed in the Table S4 in the supplemental material. *GAS1* was deleted in the *bgl2*Δ strain by chromosomal integration of an 893-bp nourseothricin (*NAT*) PCR fragment. The integrated product was PCR amplified from the pFA6a-natNT2 plasmid DNA (containing the nourseothricin marker) using primers LB-GAS1DEL-FnatNT2 and LB-GAS1DEL-RnatNT2, including part of the *GAS1* promoter and terminator regions, with the following program: 30 s at 98°C followed by 30 cycles of 10 s at 98°C, 30 s at 45°C, and 30 s at 72°C. The *bgl2*Δ strain was transformed with this construct according to the LiOAc method, and the yeast chromosomal DNA was extracted according to protocols described elsewhere (52). A 890-bp PCR fragment was amplified with primers GAS1ctrlF-PROM and natNT2REV (homologous to *GAS1* promoter and *NAT* sequences), and a 1,239-bp PCR fragment was amplified using primers natNT2FOR and GAS1ctrlR-TERM (homologous to *NAT* and *GAS1* terminator sequences) for five clones, confirming the deletion of *GAS1* gene in the *bgl2*Δ strain. Each of the five clones was able to grow on YPD medium containing geneticin (300 µg/ml) or nourseothrycin (100 µg/ml) or both. Two of the five clones were used for the entire study. The duplication of *GAS1* in these two clones has also been ruled out using the primers GAS1geneF and Gas1geneR (primers with the sequences inside *GAS1*; Table S4). GAS1verif1 and GAS1verif2 (primer sequences outside the deletion cassette) were used to verify ectopic integration of the *GAS1* deletion cassette; a 2,860-bp band was observed for the wild-type strain and a 2,000-bp band for the *gas1*Δ *bgl2*Δ mutant, confirming the presence of the nourseothricin deletion cassette at the right locus.

10.1128/mBio.00619-17.10 TABLE S4  Primers used to generate the *gas1*Δ *bgl2*Δ double mutant strain. Download TABLE S4, PDF file, 0.02 MB. Copyright © 2017 Aimanianda et al. 2017 Aimanianda et al. This content is distributed under the terms of the Creative Commons Attribution 4.0 International license.

### *S. cerevisiae* permeabilization, cell wall fractionation and solubilization, and periodate oxidation and characterization.

Permeabilization, alkali-insoluble (AI) fraction extraction from the cell wall, its solubilization using endo-β-(1,3)-glucanase, periodate oxidation-Smith degradation, high-performance anion-exchange chromatography (HPAEC/Dionex), thin-layer chromatography, and low-pressure liquid chromatography disruption of the cells to obtain cytosolic, membrane, and cell wall fractions were performed as described earlier (7). Dionex profiling was performed using PA1 and PA200 CarboPAC columns (Thermo-Fisher Scientific); the gradient run (flow rates, 1 ml/min for PA1 and 0.350 for PA200) was performed using solvent A (50 mM NaOH) and solvent B (500 mM sodium acetate–50 mM NaOH) as follows: for gradient run I, 0 to 2 min, isocratic (98% A plus 2% B), 2 to 15 min→65% A plus 35% B, 15 to 22 min→57% A plus 43% B, 22 to 23 min→100% B, and 23 to 25 min 100% B; for gradient II, 0 to 2 min, isocratic (98% A plus 2% B), 2 to 15 min →80% A plus 20% B, 15 to 20 min→57% A plus 43% B, 20 to 23 min→100% B, and 23 to 25 min 100% B; for gradient III, 0 to 2 min, isocratic (98% A plus 2% B), 2 to 15 min→65% A plus 35% B, 15 to 35 min→40% A plus 60% B, 35 to 37 min→100% B, 37 to 40 min 100% B (gradients I and II were used for the PA1 column; gradient III was used for the PA200 column). Samples were detected on a pulsed electrochemical detector (PED; for nonradiolabeled samples) or using a radiometric detector (Packard Radiomatic Flo-One, equipped with a 500-µl liquid-type cell) for ^14^C-radiolabeled samples. ^14^C-radiolabeled compounds were detected at 156 keV with a liquid scintillation flow rate of 2.0 ml/min. The representative Dionex profiles shown in the figures are reproducible, as each experiment was performed at least 3 to 5 times, sometimes over 10 times.

### Nuclear magnetic resonance (NMR) spectroscopy.

NMR spectra were acquired at 288 K on Varian Inova spectrometers operating at proton frequencies of 500 MHz and 600 MHz equipped with a triple-resonance ^1^H(^13^C/^15^N) Triax gradient probe and a cryogenically cooled triple-resonance ^1^H(^13^C/^15^N) pulsed-field gradient (PFG) probe, respectively. Sample lyophilized repeatedly in D_2_O was dissolved in 420 µl D_2_O (99.97%^2^H atoms) (Euriso-top, CEA, Saclay, France) and transferred into a 5-mm-diameter Shigemi tube (Shigemi Inc., Alison Park, USA). ^1^H chemical shifts were referenced to external DSS (4,4-dimethyl-4-silapentane-1-sulfonic acid; its methyl resonance was set to 0 ppm). ^13^C chemical shifts were then calculated from the ^1^H chemical shift and gamma ratio relative to DSS. A ^13^C/^1^H gamma ratio of 0.251449530 was used (53). The following nucleus assignment strategy was adopted. First, the proton resonances were assigned using a two-dimensional correlation spectroscopy (COSY) experiment (20). A relayed COSY experiment (RELAY) with one and two relays of 60 ms allowed us to follow connectivities from the anomeric proton up to the H4 proton of the glycosidic residues (54, 55). The intraglycosidic residue spin systems were completed by means of a total correlation spectroscopy (TOCSY) experiment with a long mixing time (100 ms) (56). Second, a ^1^H-^13^C gradient heteronuclear single-quantum correlation spectroscopy (gHSQC) experiment and a ^1^H-^13^C gHSQC-TOCSY experiment with a mixing time of 80 ms allowed achieving assignment of ^13^C chemical shifts from previously identified ^1^H resonances (57). In addition, the CH2 groups were easily identified from the ^13^C-edited gHSQC experiment. Then, analysis of ^1^H, ^1^H coupling constants (^3^*J*_H1, H2_) from a 1D and/or COSY spectrum (^1^H resolution of 0.1 Hz and 1.0 Hz, respectively) assessed the monosaccharide residue identity. Moreover, the anomeric configuration of monosaccharide residues was established from knowledge of ^3^*J*_H1, H2_ values and confirmed via the measurement of the ^1^*J*_C1, H1_ heteronuclear coupling constants in the ^1^H dimension of the gradient heteronuclear multiple-bond correlation spectroscopy (gHMBC) spectrum (^1^H resolution of 1.4 Hz) (57). Finally, glycosidic linkages were established via through-space dipolar interactions using a ^1^H, ^1^H rotating-frame Overhauser effect spectroscopy (ROESY) experiment (mixing time of 250 ms) and/or via three-bond interglycosidic ^1^H, ^13^C correlations using a ^1^H, ^13^C gHMBC experiment (long-range delay of 60 ms).

### *In situ* branching activity assay.

The assay mixture (in a total volume of 67 µl) contained 50 mM Tris-HCl buffer (pH 7.5), 0.5 mM UDP-(^14^C)glucose (specific activity, 34 nmol/125 nCi in the final reaction mixture), 0.2 mM ATP, 20 µM GTPγS, EDTA (1 mM), and permeabilized cells (5 × 10^8^ cells) at room temperature. Neosynthesized polysaccharides were precipitated overnight by the use of two volumes of cold ethanol (−20°C). The precipitate thus obtained was washed three times with water (500 µl each time) and treated twice with 500 µl of 1 M NaOH containing 0.5 M NaBH_4_ at 65°C for 1 h. The AI fraction was collected by centrifugation at 10,000 × *g* for 10 min, washed with water, neutralized using acetic acid, and subjected further to LamA digestion. Incorporation of the radioactivity was measured at each step using a Wallac 1410 liquid scintillation counter (PerkinElmer Life Sciences).

### Production of recombinant Gas1p and Bgl2p.

Recombinant Gas1p was produced using a *Pichia pastoris* expression system (12). For Bgl2p, the amino acid sequence (CAA97313.1) was back-translated into a nucleotide sequence that was codon optimized for expression in *Escherichia coli*. The gene was synthesized with flanking NdeI and XhoI restriction sites (GeneArt; Life Technologies, Inc.). The fragment was ligated into pET28a(+) expression vector (Novagen), creating a sequence with an N-terminal histidine tag. Cloning was done using *E. coli* DH5α and selection with 30 µg/ml kanamycin; the final expression vector was transformed into SHuffle T7 competent *E. coli* cells (New England Biolabs). LB medium containing selection antibiotic was inoculated with the expression strain and shaken at 200 rpm and 30°C. At an optical density of 0.8, the culture was induced with a final concentration of 1 mM isopropyl-β-d-1-thiogalactopyranoside (Sigma-Aldrich). Cells were collected after 4 h (centrifugation, 30 min at 4,000 × *g*) and suspended in 50 mM Tris-Cl (pH 8.0) containing 200 µg/ml lysozyme (Sigma-Aldrich). After 45 min, cells were disrupted with 5 sets of 10 bursts with a Sonifier cell disruptor B30 (Branson). Debris and inclusion bodies were removed by centrifugation (20 min, 16,000 × *g*), and His-tagged Bgl2p from the lysate was purified using nickel beads. An Amicon cell (Millipore) (3-kDa cutoff) was used to concentrate the sample and to change the buffer to HBS (20 mM HEPES [Sigma-Aldrich], 137 mM NaCl, pH 7.3).

### Gas1p and Bgl2p branching activity assays.

β-(1,3)-Oligosaccharides were produced as described earlier (12). To 100 µg of β-(1,3)-oligomers, 2.5 µg each of Gas1p and Bgl2p were added, separately or together, in 20 mM acetate buffer (pH 5.5) (in a total volume of 100 µl), and the reaction mixture was incubated at 37°C for different time intervals; aliquots were subjected to Dionex profiling to monitor the reaction progress. Further, LamA (2.5 µg) was added to the rest of the reaction mixture and the reaction mixture was incubated at 37°C for 20 h followed by Dionex profiling.
