# Supplementary material for: The Dual Activity Responsible for the Elongation and Branching of β-(1,3)-Glucan in the Fungal Cell Wall
Source: mBio. 2017 Jun 20;8(3):e00619-17. doi: 10.1128/mBio.00619-17 (PMC5478894; doi:10.1128/mBio.00619-17)
Supplement: TABLE S1 [file mbo003173350st1.pdf]

Supplementary Table 1: ^{1}H and ^{13}C NMR chemical shifts (ppm) and coupling constants $^3J_{H,H}$ and $^1J_{C1H1}$, Hz) for the two additional (branched) oligosaccharides released after digestion of *S. cerevisiae* AI-fraction with endo-β-(1,3)-glucanase (LamA).

**Linkages**	H1<br>$^3J_{1,2}$<br>C1	H2<br>$^3J_{2,3}$<br>C2	H3<br>$^3J_{3,4}$<br>C3	H4<br>$^3J_{4,5}$<br>C4	H5<br>$^3J_{5,6}$<br>C5	H6<br>$^2J_{6,6'}$<br>C6	H6'<br>$^3J_{5,6'}$	$^1J_{C1H1}$
**Branched trisaccharides (major)**								
**β-Glc-(1-6)-**<br>L	4.510<br>7.3<br>105.42	3.307<br>8.9<br>75.79	3.482<br><br>78.54	3.380<br><br>72.24	3.436<br><br>78.21	3.723<br><br>63.33	3.906	
**-6)-β-Glc-(1-3)-**<br>G	4.704<br>7.7<br>105.69	3.364<br>8.1<br>75.96	3.522<br>8.2<br>78.11	3.477<br>9.6<br>72.19	3.651<br>6.4<br>77.41	**3.855**<br>11.9<br>**71.43**	**4.206**	
**-3)-β-Glc**<br>I	4.667<br>8.1<br>98.38	3.425<br>9.1<br>76.35	3.734<br>7.9<br>**88.00**	3.487<br>7.9<br>70.86	3.506<br><br>78.27	3.704<br>11.5<br>63.36	3.888	
**β-Glc-(1-6)-**<br>L	4.510<br>7.3<br>105.42	3.307<br>8.9<br>75.79	3.482<br><br>78.54	3.380<br><br>72.24	3.436<br><br>78.21	3.723<br><br>63.33	3.906	
**-6)-β-Glc-(1-3)-**<br>H	4.687<br>7.7<br>105.72	3.364<br>8.1<br>75.96	3.522<br>8.2<br>78.11	3.477<br>9.6<br>72.19	3.651<br>6.4<br>77.41	**3.855**<br>11.9<br>**71.43**	**4.206**	
**-3)-α-Glc**<br>A<br><br>or<br>**-3)-α-Glc**<br>B	5.231<br>3.3<br>94.72<br><br>5.222<br>3.3<br>94.72	3.708<br>8.9<br>73.59<br><br>3.713<br>8.4<br>73.84	3.865<br>9.4<br>**85.89**<br><br>3.907<br>9.4<br>**84.81**	3.520<br>9.1<br>70.86<br><br>3.507<br>9.7<br>70.77	3.880<br><br>73.84<br><br>3.854<br><br>73.84	3.772<br><br>63.15<br><br>3.772<br><br>63.15	3.806<br><br><br><br>3.806	 170.3<br><br><br><br>170.3
**Branched tetrasaccharides (minor)**								
**β-Glc-(1-3)-**<br>F	4.741<br>~ 8<br>105.48	3.348<br>8.8<br>76.10	3.517<br><br>78.22	3.392<br>~ 9<br>72.25				
**3)-β-Glc-(1-6)-**<br>K	4.522<br>7.5<br>105.36	3.492<br>7.9<br>75.68	3.746<br><br>**86.69**	3.498<br><br>70.77	3.486<br><br>78.19	3.711<br><br>63.27	3.897	
**-6)-β-Glc-(1-3)-**<br>H	4.687<br>7.7<br>105.72	3.364<br>8.1<br>75.96	3.522<br>8.2<br>78.11	3.477<br>9.6<br>72.19	3.651<br>6.4<br>77.41	**3.855**<br>11.9<br>**71.43**	**4.206**	
**-3)-α-Glc**<br>A<br><br>or<br>**-3)-α-Glc**<br>B	5.231<br>3.3<br>94.72<br><br>5.222<br>3.3<br>94.72	3.708<br>8.9<br>73.59<br><br>3.713<br>8.4<br>73.84	3.865<br>9.4<br>**85.89**<br><br>3.907<br>9.4<br>**84.81**	3.520<br>9.1<br>70.86<br><br>3.507<br>9.7<br>70.77	3.880<br><br>73.84<br><br>3.854<br><br>73.84	3.772<br><br>63.15<br><br>3.772<br><br>63.15	3.806<br><br><br><br>3.806	 170.3<br><br><br><br>170.3
**β-Glc-(1-3)-**<br>F	4.741<br>~ 8	3.348<br>8.8	3.517	3.392<br>~ 9				

	105.48	76.10	78.22	72.25				
**3)-β-Glc-(1-6)-**<br>K	4.522<br>7.5<br>105.36	3.492<br>7.9<br>75.68	3.746<br><br>**86.69**	3.498<br><br>70.77	3.486<br><br>78.19	3.711<br><br>63.27	3.897	
**-6)-β-Glc-(1-3)-**<br>G	4.704<br>7.7<br>105.69	3.364<br>8.1<br>75.96	3.522<br>8.2<br>78.11	3.477<br>9.6<br>72.19	3.651<br>6.4<br>77.41	**3.855**<br>11.9<br>**71.43**	**4.206**	
**-3)-β-Glc**<br>I	4.667<br>8.1<br>98.38	3.425<br>9.1<br>76.35	3.734<br>7.9<br>**88.00**	3.487<br>7.9<br>70.86	3.506<br><br>78.27	3.704<br>11.5<br>63.36	3.888	
