# Supplementary material for: The Dual Activity Responsible for the Elongation and Branching of β-(1,3)-Glucan in the Fungal Cell Wall
Source: mBio. 2017 Jun 20;8(3):e00619-17. doi: 10.1128/mBio.00619-17 (PMC5478894; doi:10.1128/mBio.00619-17)
Supplement: TABLE S2 [file mbo003173350st2.pdf]

Supplementary Table 2: ^{1}H and ^{13}C NMR chemical shifts (ppm) and coupling constants ($^{3}J_{H,H}$ and $^{1}J_{C1H1}$, Hz) for the "branched" fraction obtained after β-(1,3)-glucanase digestion of the AI-fraction of the wild-type *S. cerevisiae* cell-wall.

**Linkages**	**H1**<br>**$^3J_{1,2}$**<br>**C1**	**H2**<br>**$^3J_{2,3}$**<br>**C2**	**H3**<br>**$^3J_{3,4}$**<br>**C3**	**H4**<br>**$^3J_{4,5}$**<br>**C4**	**H5**<br>**$^3J_{5,6}$**<br>**C5**	**H6**<br>**$^2J_{6,6'}$**<br>**C6**	**H6'**<br>**$^3J_{5,6'}$**	**$^1J_{C1H1}$**
**A**<br>-3)-α-Glc	5.231<br>3.3<br>94.72	3.701<br>8.9<br>73.59	3.865<br>9.4<br>**85.89**	3.520<br>9.1<br>70.86	3.880<br><br>73.84	3.772<br><br>63.15	3.806	170.3
**B**<br>-3)-α-Glc	5.222<br>3.3<br>94.72	3.713<br>8.4<br>73.84	3.907<br>9.4<br>**84.81**	3.507<br>9.7<br>70.77	3.854<br><br>73.84	3.772<br><br>63.15	3.806	170.3
**C**<br>3)-β-Glc-(1-3)-	4.776<br>7.6 and 8.1<br>105.26	3.552<br>8.6<br>76.00	3.778<br>8.9<br>**86.78**	3.498<br><br>70.74	3.486<br><br>78.22	3.711<br><br>63.31	3.897	
**D**<br>3)-β-Glc-(1-3)-	4.756<br>~ 8<br>105.48	3.554<br>8.6<br>76.00	3.786<br>8.9<br>**86.78**	3.498<br><br>70.7	3.486<br><br>78.22	3.711<br><br>63.31	3.900	
**E**<br>β-Glc-(1-3)-	4.751<br>8.1<br>105.48	3.351<br>8.6<br>76.10	3.521<br>8.9<br>78.18	3.392<br>~ 9<br>72.23	3.457<br><br>78.68	3.711<br><br>63.43	3.900	165.8
**F**<br>β-Glc-(1-3)-	4.741<br>~ 8<br>105.48	3.348<br>8.8<br>76.10	3.517<br><br>78.22	3.392<br>~ 9<br>72.25				
**G**<br>-6)-β-Glc-(1-3)-	4.704<br>7.7<br>105.69	3.364<br>8.1<br>75.96	3.522<br>8.2<br>78.11	3.477<br>9.6<br>72.19	3.651<br>6.4<br>77.41	**3.855**<br>11.9<br>**71.43**	**4.206**	
**H**<br>-6)-β-Glc-(1-3)-	4.687<br>7.7<br>105.72	3.364<br>8.1<br>75.96	3.522<br>8.2<br>78.11	3.477<br>9.6<br>72.19	3.651<br>6.4<br>77.41	**3.855**<br>11.9<br>71.43	**4.206**	
**I**<br>-3)-β-Glc	4.667<br>8.1<br>98.38	3.425<br>9.1<br>76.35	3.734<br>7.9<br>**88.00**	3.487<br>7.9<br>70.86	3.506<br><br>78.27	3.704<br>11.5<br>63.36	3.888	
**J**<br>-3)-β-Glc	4.645<br>8.1<br>98.55	3.429<br>7.8<br>76.52	3.742<br>8.4<br>**86.99**	3.487<br>7.9<br>70.88	3.527<br><br>78.13	3.704<br>11.5<br>63.36	3.888	
**K**<br>3)-β-Glc-(1-6)-	4.522<br>7.5<br>105.36	3.492<br>7.9<br>75.68	3.746<br><br>**86.69**	3.498<br><br>70.77	3.486<br><br>78.19	3.711<br><br>63.27	3.897	
**L**<br>β-Glc-(1-6	4.510<br>7.3<br>105.42	3.307<br>8.9<br>75.79	3.482<br><br>78.54	3.380<br><br>72.24	3.436<br><br>78.21	3.723<br><br>63.33	3.906	

major branched and linear oligosaccharides:<br>β-Glc-(1-6)-β-Glc-(1-3)-α,β-Glc (L1-6G1-3I and L1-6H1-3A or B)<br>β-Glc-(1-3)-β-Glc-(1-3)-β-Glc (E1-3CD1-3J)<br>β-Glc-(1-3)-α-Glc (E1-3B)
minor branched oligosaccharides<br>β-Glc-(1-3)-β-Glc-(1-6)-β-Glc-(1-3)-α,β-Glc (F1-3K1-6H1-3A or B and F1-3K1-6G1-3I)
