# Supplementary material for: The Dual Activity Responsible for the Elongation and Branching of β-(1,3)-Glucan in the Fungal Cell Wall
Source: mBio. 2017 Jun 20;8(3):e00619-17. doi: 10.1128/mBio.00619-17 (PMC5478894; doi:10.1128/mBio.00619-17)
Supplement: TABLE S3 [file mbo003173350st3.pdf]

Supplementary Table 3: *S. cerevisiae* mutant strains analyzed for branched oligosaccharides

**Gene deleted**	**Function✯**	**Branching***
BGL2	Glycosyltransferase	Slightly decreased (~15%)
GAS1		**Decrease (~70%)**
CRH1	Chitin transglycosylase	Normal
CRH2		
CHS3	Chitin synthase	Normal
DCW1	Putative mannanase, GPI-anchored membrane protein	Normal
DFG5		
ECM33	GPI-anchored protein of unknown function	Normal
KRE1	β-(1,6)-glucan biosynthesis	Normal
KRE5		
KRE6		
KRE9		
APM1	β-(1,6)-glucan biosynthesis related (Homologous and physical binding)	Normal
CNB1		
SKN1		
KEG1		
KNH1		
KNR4		
KIN2		
LAS17		
SLA1		

*Comparison with the wild type (BY4741) strain; branching percent was calculated from the peak areas in the DIONEX profiles using the ratio, area occupied by the branched trimer + tetramer peaks and the total area occupied by all the peaks.

✯http://www.yeastgenome.org/
