# Supplementary figures and images for: The Dual Activity Responsible for the Elongation and Branching of β-(1,3)-Glucan in the Fungal Cell Wall
Source: mBio. 2017 Jun 20;8(3):e00619-17. doi: 10.1128/mBio.00619-17 (PMC5478894; doi:10.1128/mBio.00619-17)

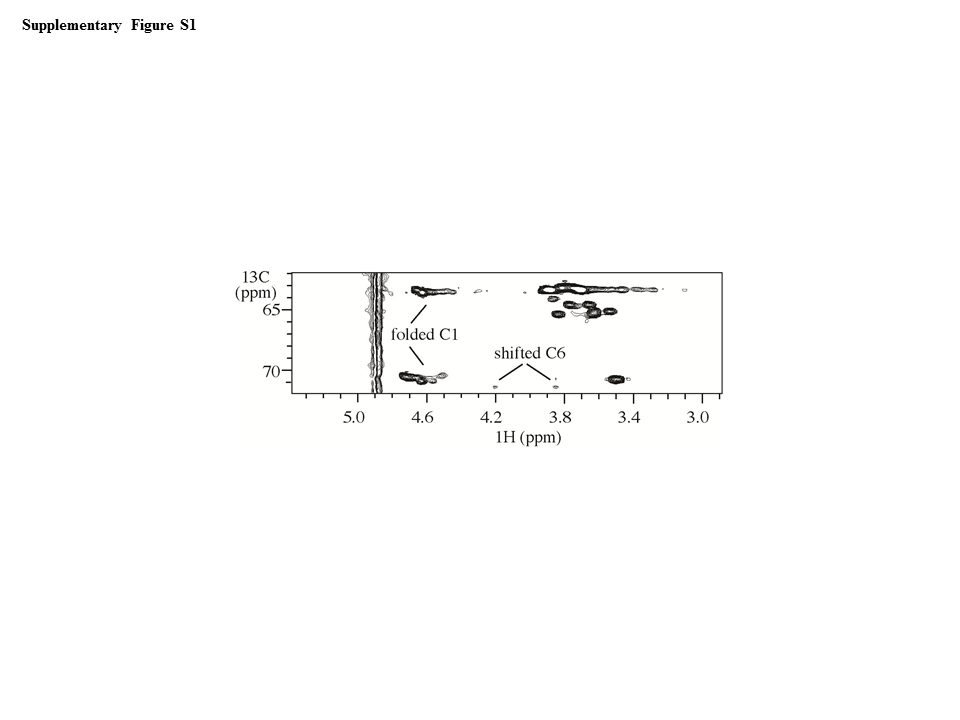

Supplement: FIG S1 [file mbo003173350sf1.tif]

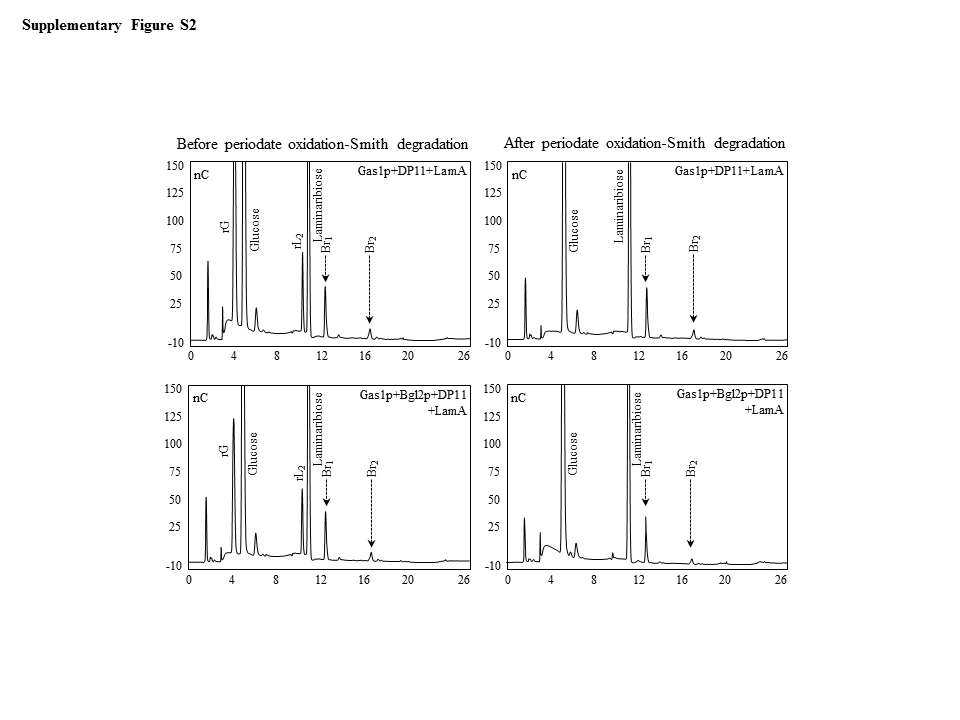

Supplement: FIG S2 [file mbo003173350sf2.tif]

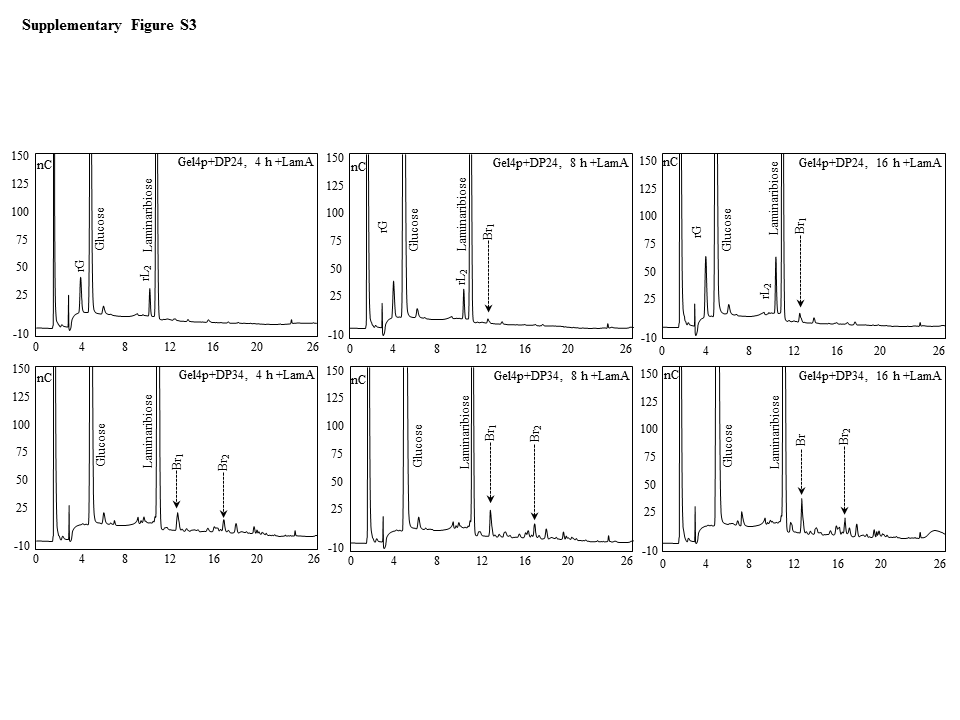

Supplement: FIG S3 [file mbo003173350sf3.tif]

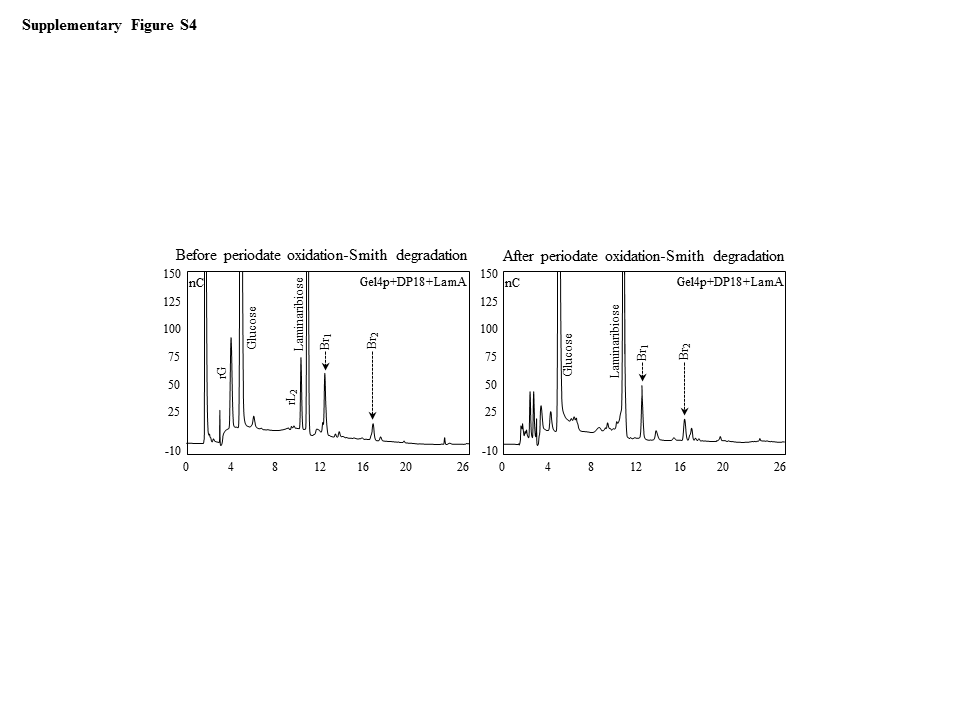

Supplement: FIG S4 [file mbo003173350sf4.tif]

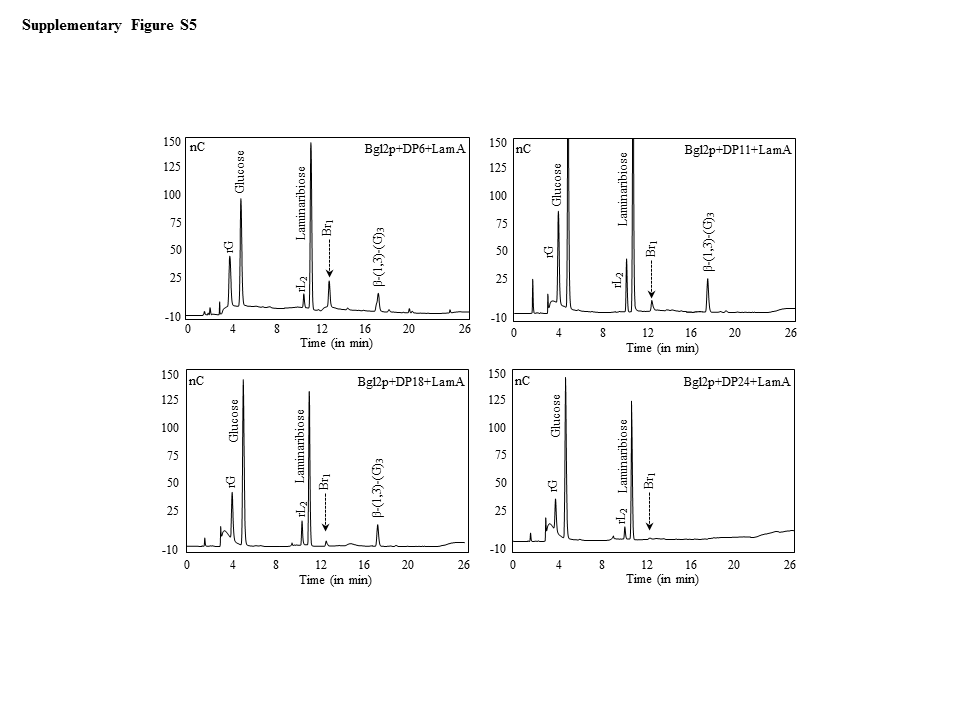

Supplement: FIG S5 [file mbo003173350sf5.tif]

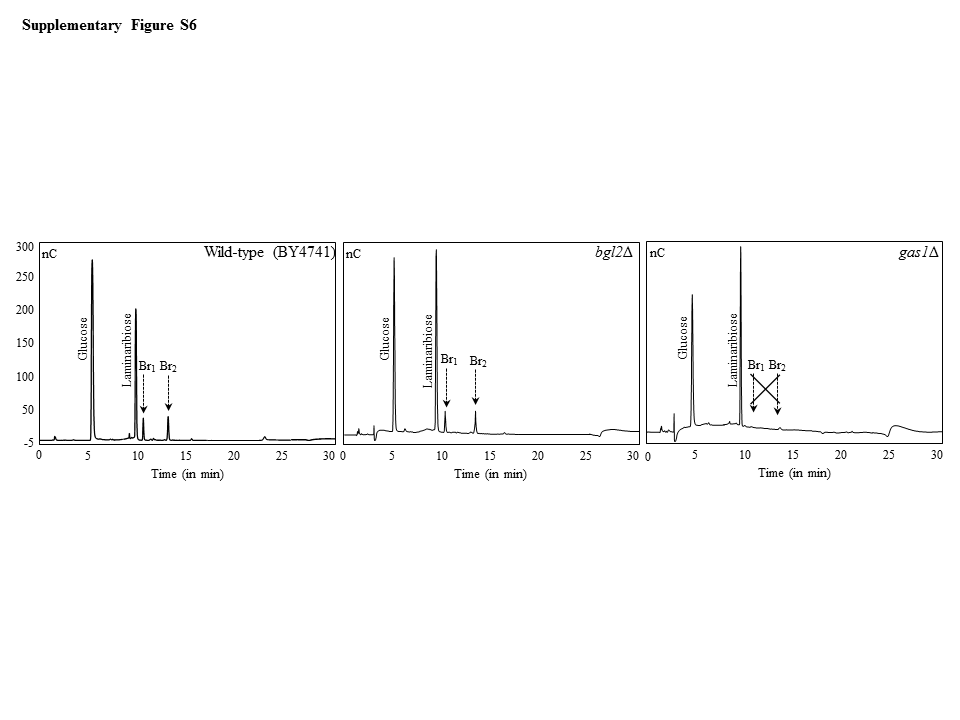

Supplement: FIG S6 [file mbo003173350sf6.tif]
