# Supplementary material for: The Dual Activity Responsible for the Elongation and Branching of β-(1,3)-Glucan in the Fungal Cell Wall
Source: mBio. 2017 Jun 20;8(3):e00619-17. doi: 10.1128/mBio.00619-17 (PMC5478894; doi:10.1128/mBio.00619-17)
Supplement: TABLE S4 [file mbo003173350st4.pdf]

Supplementary Table 4: Primers used to generate *gas1*Δ*bgl2*Δ double mutant strain

**Primers used to delete *GAS1* in the *bgl2*Δ background**		
FOR	LB-GAS1DEL-FnatNT2	AAAGTCTGATAAAACAAAAACAACAAACACAGCTAAATCTCAACAGTTGAATTGTCCCCACGC
REV	LB-GAS1DEL-RnatNT2	AAGATACCATACCTTATCGAGTTATTATGTATGTGTCGAAGCTTTGTCACTTTAAAATTTGTATACAC
**Primers used to verify the double *gas1*Δ*bgl2*Δ deletion strain generated**		
FOR	GAS1ctrlF-PROM	TAAAGCGAGCTGGTGCCTAT
REV	natNT2 (REV)	CGAGTACGAGATGACCACGA
FOR	natNT2 (FOR)	ACTGGATGGGTCCTTCACC
REV	GAS1ctrlR-TERM	TCAACAGAGAATGCCAGTGC
GAS1geneF		CAGCTCCAGCCACCTCTATC
GAS1geneR		ACGTCATCGGAAACAACACAA
GAS1verif1		TGACTCGAAAGCAGAGAGCA
GAS1verif2		TCAACAGAGAATGCCAGTGC
